# Multistep process of FUS aggregation in the cell cytoplasm involves RNA-dependent and RNA-independent mechanisms

**DOI:** 10.1093/hmg/ddu243

**Published:** 2014-05-19

**Authors:** Tatyana A. Shelkovnikova, Hannah K. Robinson, Joshua A. Southcombe, Natalia Ninkina, Vladimir L. Buchman

**Affiliations:** 1 School of Biosciences, Cardiff University, Museum Avenue, CardiffCF10 3AX, UK,; 2 Institute of Physiologically Active Compounds Russian Academy of Sciences, 1 Severniy proezd, Chernogolovka 142432, Moscow Region, Russian Federation and; 3 Institute of General Pathology andPathophysiology of Russian Academy of Medical Science, 8 Baltijskaya str, Moscow 125315, Russian Federation

## Abstract

Fused in sarcoma (FUS) is an RNA-binding protein involved in pathogenesis of several neurodegenerative diseases. Aggregation of mislocalized FUS into non-amyloid inclusions is believed to be pivotal in the development of cell dysfunction, but the mechanism of their formation is unclear. Using transient expression of a panel of deletion and chimeric FUS variants in various cultured cells, we demonstrated that FUS accumulating in the cytoplasm nucleates a novel type of RNA granules, FUS granules (FGs), that are structurally similar but not identical to physiological RNA transport granules. Formation of FGs requires FUS N-terminal prion-like domain and the ability to bind specific RNAs. Clustering of FGs coupled with further recruitment of RNA and proteins produce larger structures, FUS aggregates (FAs), that resemble but are clearly distinct from stress granules. In conditions of attenuated transcription, FAs lose RNA and dissociate into RNA-free FUS complexes that become precursors of large aggresome-like structures. We propose a model of multistep FUS aggregation involving RNA-dependent and RNA-independent stages. This model can be extrapolated to formation of pathological inclusions in human FUSopathies.

## INTRODUCTION

Studies of RNA-binding proteins TAR DNA binding protein of 43 kDa (TDP-43) and fused in sarcoma (FUS) were given an extra dimension when these proteins were identified as causative factors for a number of degenerative diseases, primarily amyotrophic lateral sclerosis (ALS) and frontotemporal lobar degeneration (FTLD) (reviewed in 1). Aggregation of these proteins followed by the formation of intracellular inclusions and the development of respective proteinopathy is believed to be a crucial event in the onset and progression of pathology. Two major consequences of abnormal FUS compartmentalization can be envisaged: loss of essential functions in the nucleus, and gain of toxic function(s) in the cytoplasm. Currently available *in vivo* data support both mechanisms (reviewed in 2) since in some studies neurotoxicity upon expression of mutant FUS variants was observed (3–8) and co-expression of normal FUS could not rescue the toxicity of mutant FUS (9), while in other studies loss of FUS caused neuronal deficits (4,8,10,11). However, results obtained in the majority of studies carried out in available *in vivo* models strongly suggest that mislocalized FUS can cause cell dysfunction independently of the effects of its reduced nuclear levels. FUS is an established component of neuronal RNA transport granules (12) and can be sequestered into stress-induced stress granules (SGs) (13). The latter ability is greatly enhanced by mutations affecting the nuclear localization signal (NLS) and consequent retention of the protein in the cytoplasm (14–17). Abundance of RNA granules is characteristic of neurons, which require large distance transport of specific proteins involved in local translation in axons, dendrites and synaptic terminals. Unsurprisingly, many of these proteins are to a various extent linked to pathology in humans (reviewed in 18). The ability of mislocalized FUS to aggregate spontaneously in the cytoplasm of cultured cells and even in *in vivo* models with the formation of granule-like structures has been repeatedly reported (9,19–22). It is likely that similar structures are formed in neuronal and glial cells at the early stages of pathology development.

Recently, we have demonstrated that engineered FUS variants lacking the ability to efficiently bind target RNAs and be sequestered in SGs are extremely prone to aggregate and form large inclusions in cellular and transgenic mouse models (23,24). These irreversible FUS aggregates (FAs) display different features from granule-like structures formed in the cytoplasm of cultured cells by ALS-associated FUS variants carrying mutations in the nuclear localization signal. We proposed that the latter structures are organized similarly to physiological RNP granules but in particular conditions might be transformed into structurally different final products of FUS aggregation, resembling inclusions typical for FUSopathies. To test this, we characterized granules formed by ALS-associated FUS variants accumulating in the cells cytoplasm and their transformations under conditions of stress and attenuated transcription.

## RESULTS

### Cytoplasmic FUS spontaneously aggregates in cultured cells in a concentration-dependent manner

**Figure 1.**
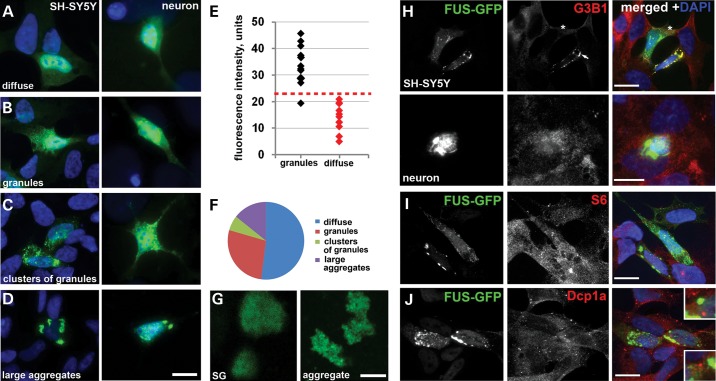
FUS with disrupted nuclear localization signal accumulates in the cytoplasm and forms granule-like aggregates in a concentration-dependent manner. Neuroblastoma SH-SY5Y cells, COS7 cells or mixed primary neuronal-glial cultures were transiently transfected with an expression plasmid encoding the GFP-tagged FUS variant bearing R522G amino acid substitution in the NLS. The protein either displays diffuse distribution (**A**) or is found in multiple granule-like microaggregates *(FGs,* **B**) that in some cells form clusters (**C**) or large compact aggregates (*FAs*, **D**). (**E**) Aggregation of FUS in the cytoplasm is concentration dependent. Arbitrary units of fluorescence intensity in the cytoplasm of individual cells (24 h after transfection with GFP-FUS R522G expression plasmid) with diffuse only distribution or with already formed granules are plotted. (**F**) Proportion of cells with various types of FUS R522G distribution in the population of expressing cells. (**G**) High-resolution confocal images of FUS R522G-positive stress granules (SG) formed in low-expressing COS7 cells upon sodium arsenite treatment and FAs of similar size formed by this protein in naïve COS7 cells (aggregate). (**H**) Only a fraction of FAs formed in naïve SH-SY5Y cells or primary neurons are positive for a SG marker G3B1 (arrow), while in others FAs do not contain this protein (cell marked with an asterisk). (**I**) Ribosomal protein S6 is not accumulated in FGs or FAs. (**J**) P-bodies detected by anti-Dcp1a staining do not occur in the immediate vicinity to FAs (compare to insets in Supplementary Material, Fig. S3). Scale bars (G), 2 µm, all other panels—10 µm.

Consistent with the results of previous studies (15,16,19,25), GFP-tagged FUS variants rendered cytoplasmic by the introduction of mutations or truncations abrogating nuclear import were diffusely distributed in the cytoplasm of SH-SY5Y neuroblastoma cells or primary hippocampal neurons (Fig. 1A, Supplementary Material, Fig. S1A). However, after reaching a certain concentration threshold (as measured by fluorescence intensity, Fig. 1E), these FUS variants aggregated forming either multiple small granule-like microaggregates (*FUS granules*, FGs) (Fig. 1B), which subsequently assembled into loose clusters (Fig. 1C), or larger, more compact aggregates (Fig. 1D). In the population of cells with non-diffuse FUS distribution, those with FGs are more abundant than cells with other two types of structures (Fig. 1F). Since granule clusters and large aggregates clearly represent the same structure at early and late stages of its formation, they will be further combined into one group and referred to as *FAs*. The same pattern of FUS aggregation was found in HEK293, MCF7 and COS7 cells (Supplementary Material, Fig. S1B and data not shown). Flag-tagged cytoplasmic FUS variants behaved similarly to GFP-tagged ones (Supplementary Material, Fig. S2A,B); therefore, we used GFP-labelled proteins in all subsequent studies. In contrast, we did not observe nucleation of similar granules or aggregates by TDP-43 mislocalized to the cytoplasm due to the deletion of its NLS despite high cytoplasmic levels of the protein in transfected cells (Supplementary Material, Fig. S2C and D).

We observed significant differences in organization and dynamics of the structures formed by ectopically accumulated FUS and induced SGs. High-magnification images clearly demonstrated that mature SGs present in stressed neuroblastoma SH-SY5Y cells occur as distinct compact structures, whereas FAs of similar size are non-compact, irregular shaped constellations of small round granules (Fig. 1G). The assembly of SGs in heat shock- or sodium arsenite-stressed cells is very rapid with the stage of small precursors barely detectable, in contrast, FGs can persist in the cell for at least several hours without clustering as can be judged from a significant proportion of cells containing these structures (Fig. 1F). While further fusion is not typical for mature SGs, FAs can merge, giving rise to larger aggregates, sometimes one or two per cell (Fig. 1D, Supplementary Material, Fig. S1 and Video S1). Furthermore, a fraction of FAs are negative or only weakly positive for established SG markers, G3B1 and TIAR (Fig. 1H; Supplementary Material, Fig. S3A), which indicates that their integrity does not require SG proteins. Ribosomal protein S6—the component of small ribosomal subunit—is a usual component of SGs (26), but this protein was not detected in FGs or FAs (Fig. 1I), confirming that stalled pre-initiation complexes are not major constituents of these structures. Another type of RNP granules, processing bodies (P-bodies), is known to occur in close proximity to SGs in stressed cultured cells (27). Using anti-Dcp1a staining, we observed at least one or two P-bodies docked with almost every SG in stressed SH-SY5Y cells (Supplementary Material, Fig. S3B). However, P-body numbers were significantly reduced in non-stressed cells possessing FGs and/or FAs (see below) and in those that retained P-bodies no such docking was evident between P-bodies and FAs (Fig. 1J, compare insets in this figure and in Supplementary Material, Fig. S3B).

### RNA is an important structural component of FAs required for their integrity and sequestration of SG-associated proteins

**Figure 2.**
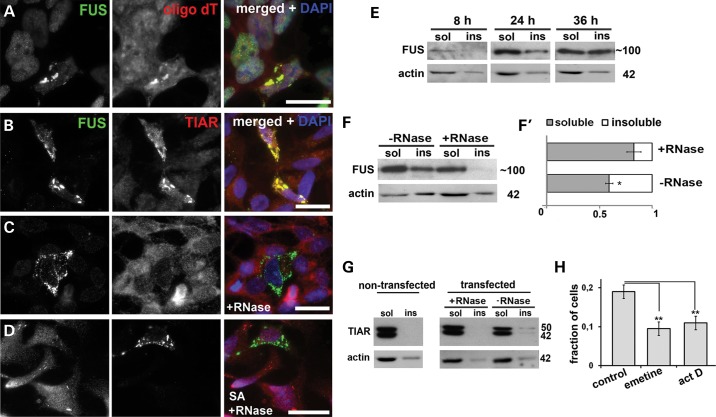
RNA is an integral component of FAs and is required for recruitment of stress granule proteins to these structures. (**A**) FAs formed by GFP-FUS R522G in SH-SY5Y cells are enriched in polyA mRNA as revealed by RNA-FISH with fluorescently labeled oligo(dT) probe. (**B–D**) Some FAs are strongly positive for TIAR (B), but this protein can be removed from all aggregates by RNase treatment on cover slips after mild methanol fixation (C) while sodium arsenite (SA)-induced SGs remain TIAR positive following this treatment (D). (**E**) Western blots show that FUS R522G progressively accumulates in Triton X-100 insoluble (ins) fraction of transfected SH-SY5Y cells. (**F** and **F**′) RNase treatment of cell lysates prior to fractionation shifts GFP-FUS R522G protein to soluble (sol) fraction. Equal proportions of soluble and aggregated fractions were analysed on western blots. (**G**) TIAR is found only in soluble fraction in non-transfected cells, is partially recruited into insoluble fraction in cells expressing FUS R522G but can be re-solubilized by digestion of lysates with RNase A prior to fractionation. (**H**) Treatment with a translational inhibitor emetine or a global transcriptional repressor actinomycin D (act D) for 2 h results in disintegration of a significant proportion of FAs and consequently, reduction of a fraction of GFP-FUS R522G-expressing cells bearing these structures. For western blots, sizes of proteins (in kDa) are shown. Bar charts in (F′) and (H) show means ± SEM, *P < 0.05. Scale bars, 15 µm.

Although our data strongly suggested that FAs are distinct from SGs, a significant fraction of these structures displayed positive staining for SG markers (Figs 1H and 2B). This can be explained by secondary recruitment of SG proteins via binding to RNA molecules trapped in the FAs by interaction with RNA-binding domains of FUS. To test this possibility, we first demonstrated by *in situ* hybridization with oligo(dT) probe that polyadenylated transcripts are integral components of FGs (Fig. 2A). Further, we performed RNase A digestion of FAs on cover slips after mild methanol fixation. This treatment abolished TIAR staining of all FAs preserved on the cover slip (Fig. 2, compare panels B and C). Oxidative stress-induced SGs could still be detected by anti-TIAR antibody after RNase digest (Fig. 2D), indicating that our findings are not due to impaired staining under used conditions. We therefore conclude that TIAR is weakly associated with FAs via interaction with RNA; presumably, TIAR is present on the surface of FAs and can be readily removed by RNase treatment even after methanol fixation.

However, in *fixed* cells, FAs themselves were resistant to RNase treatment (Fig. 2C and D). Therefore, to further assess a role of RNA in the integrity of FAs, we prepared a fraction enriched in these structures from *unfixed* GFP-FUS R522G transfected cells. The protein accumulated in Triton X-100-insoluble fraction with time (Fig. 2E), and when lysates of cells collected 24 h post-transfection were treated with RNase A before fractionation, a dramatic shift of FUS from insoluble to soluble fraction was observed (Fig. 2F and F′). In agreement with immunofluorescence data, a small amount of TIAR was present in the insoluble fraction but its solubility was completely restored by RNase A treatment (Fig. 2G).

The integrity of FAs depended on the availability of transcripts running off polysomes, since their stalling in pre-initiation complexes by treatment of FUS R522G transfected cells with a translational inhibitor emetine significantly reduced the fraction of cells with detectable FAs (Fig. 2H). Newly synthesized transcripts also contribute to aggregate integrity because a similar decrease was observed after inhibition of transcription with actinomycin D (Fig. 2H).

### N-terminal prion-like domain and the ability to bind to a specific pool of RNAs are both required for FUS aggregation in the cell cytoplasm

**Figure 3.**
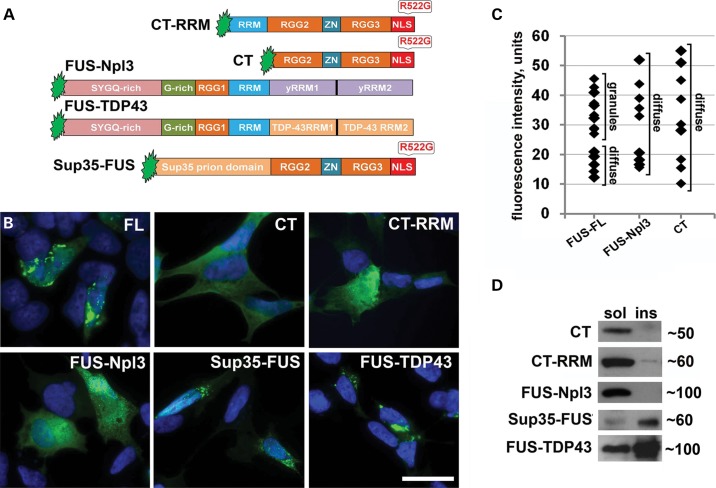
N-terminal prion-like domain and the ability to bind specific RNAs are required for the formation of FAs in SH-SY5Y cells. (**A**) Domain structure of truncated and chimera proteins used in the study. In FUS-Npl3, major RNA-binding domains of FUS (amino acids 360–526) were replaced by RRMs (yRRM1 and yRRM2) of the yeast RNA-binding protein Npl3. In FUS-TDP-43 chimera, the same FUS domains were substituted by RRM1 and RRM2 of TDP-43 protein. In Sup-35-FUS, N-terminal part (amino acids 1–359) of FUS comprising the prion-like domain was replaced by the prion domain of the yeast protein Sup35 (amino acids 1–125). (**B**) C-terminal FUS fragments (CT and CR-RRM) and FUS-Npl3 chimera display only diffuse distribution in the cytoplasm of transfected cells, whereas full-length FUS or FUS-TDP-43 and Sup35-FUS chimeras can form typical FAs. (**C**) Measurement of GFP fluorescence intensity in the cell cytoplasm showed that in many transfected cells with diffuse distribution of GFP-fused CT and FUS-Npl3, these proteins accumulate at the level above the threshold for aggregation of full-length FUS. Fluorescence intensity was measured 24 h post-transfection and values (in arbitrary units) for individual cells were plotted. (**D**) Western blot analysis of proteins in fractionated lysates of transfected cells demonstrates that full-length FUS, FUS-TDP-43 and Sup-35-FUS are recovered in Triton X-100 insoluble fraction, while CT, CT-RRM and FUS-Npl3 are essentially soluble. Scale bar, 15 µm.

FUS bears in its N-terminal part a strong prion-like domain (28,29), which was shown to be crucial for the protein localization in RNP particles (30). It is feasible that this domain is also involved in the formation and/or growth of FAs along with RNA-binding domains of the protein. To test this, we used CT and CT-RRM constructs (Fig. 3A), comprising the main RNA-binding domains of FUS but missing the entire N-terminal part; the R522G mutation was introduced to the NLS of these proteins to ensure cytoplasmic accumulation (Fig. 3A). No aggregation of these two proteins was detected, even in cells with high level of the ectopic protein expression that readily triggered aggregation of full-length R522G FUS (Fig. 3B and C) and the protein was present only in the detergent-soluble fraction (Fig. 3D). The prion domain of an yeast protein Sup35 does not form aggregates when expressed in neuroblastoma cells (data not shown), but its addition to the CT fragment of FUS (Sup35-FUS construct, Fig. 3A) was sufficient to restore aggregation capacity—like full-length FUS this fusion protein formed granules and large aggregates, and was recovered in insoluble fraction (Fig. 3B and D). To establish if RNA-binding specificity also contributes to FUS aggregation, we created two constructs encoding chimeric proteins in which C-terminal RNA-binding domains of FUS were replaced by non-homologous RNA recognition motifs, either from a related human protein, TDP-43, or an evolutionary distant protein, yeast Npl3 (Fig. 3A). FUS-TDP-43 chimeric protein readily aggregated in the cytoplasm, appeared in detergent-insoluble fraction (Fig. 3D) and formed structures resembling aggregates in cells expressing FUS R522G (Fig. 3B). In contrast, FUS-Npl3 chimeric protein was found only in soluble fraction (Fig. 3D) and its distribution was exclusively diffuse regardless of expression levels (Fig. 3B and C). Therefore, even formation of FGs, the process initiating FUS aggregation in the cytoplasm, requires both the prion-like activity of its N-terminal domains and binding to a specific pool of RNAs.

### FGs are a novel type of RNA granule

**Figure 4.**
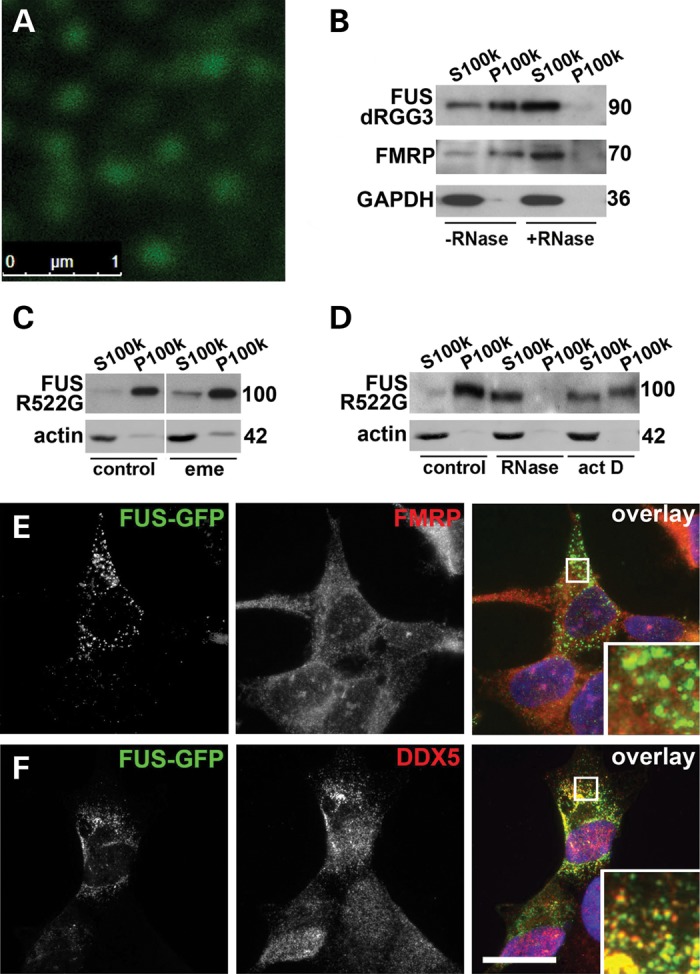
FGs nucleated by cytoplasmically accumulated FUS are built on newly synthesized RNA and resemble but not identical to RNA transport granules. (**A**) High-magnification confocal image of FGs formed by GFP-FUS R522G in the cytoplasm of SH-SY5Y cells. (**B**) FGs are RNase-sensitive detergent-resistant structures. Western blot of proteins from a granule-enriched fraction (P100K) and soluble (S100K) isolated in the presence or absence of RNase A (see Materials and Methods) from cells transfected with GFP-fused FUS dRGG3. The membrane was reprobed with antibodies against FMRP, a core protein of neuronal RNA transport granules, and soluble GAPDH protein. Novel transcripts (**D**) but not mRNAs from polysomes (**C**) contribute to the stability of FGs. SH-SY5Y cells expressing another GFP-fused cytoplasmic variant, FUS R522G, were treated with translational elongation inhibitor emetine (C) or transcriptional repressor actinomycin D (D) for 4 h prior to lysis and fractionation to granule-enriched (100 000 g pellet, P100k) and soluble (100 000 g supernatant, S100k) fractions. RNase A-treated sample was processed in parallel as an internal positive control. FGs are negative for FMRP (**E**) but a subset of these granules is DDX5-positive (**F**). In (B–D), three times more of P100k fraction relative to S100k fraction was loaded; for western blots, sizes of proteins (in kDa) are shown. Scale bars, 15 µm.

In cells expressing cytoplasmic variants of FUS, FGs appeared to be the smallest FUS-positive particles detected using conventional microscopy. High-resolution confocal microscopy revealed that they are quite uniform in size with average diameter of ∼150–200 nm (Fig. 4A). FUS is an established component of kinesin-associated RNA transport granules in neurons (12), can be recruited to dendrites via microtubules and actin filaments (31,32) possibly also as a part of macromolecular complexes and recently was found in APC-RNA granules (22). RNA transport granules measure 100–200 nm in diameter and are non-ionic detergent-resistant RNase sensitive complexes. To test if FGs display the same features, we isolated granule-enriched fraction from RNase A-treated or untreated lysates of cells expressing cytoplasmic forms of the protein, either FUS R522G or FUS lacking a portion of RGG3 (dRGG, corresponding to a familial ALS-associated mutant variant G466VfsX14). To achieve this, high-speed centrifugation of the FAs-depleted supernatant in Triton X-100 containing buffer was employed as described in Materials and Methods. RNase treatment efficiently dissipated both FGs and RNA granules, as can be judged by dramatic increase of FUS solubility observed in treated samples, mirroring the behaviour of an established component of RNA granules, fragile X mental retardation protein (FMRP) (Fig. 4B). To establish if FGs are nucleated by recruiting new transcripts upon their transport to the cytoplasm or rather by transcripts detaching from polysomes, we treated FUS R522G expressing cells with actinomycin D to globally inhibit transcription or emetine to stall mRNA on polysomes. FGs were hardly sensitive to 4 h emetine exposure (Fig. 4C), which readily dissipated FAs (Fig. 2H), but 4 h actinomycin D treatment caused significant transition of FUS from insoluble FGs fraction to Triton X-100 soluble supernatant fraction (Fig. 4D). Consistently, we observed that FGs often accumulated along nuclear periphery (Fig. 1B) where nucleation is most likely to occur upon interaction of FUS molecules with novel mRNAs leaving the nucleus.

Does FUS incorporate into preexisting, endogenous RNA granules or rather these granules are a distinct type of structure nucleated *de novo* by abnormally accumulated protein? We used known neuronal RNA granule markers to check for possible overlap with FGs. We did not detect any co-staining of FGs with FMRP, a principal structural protein of neuronal RNA granules (Fig. 4E, see also Supplementary Material, Fig. S4A for the control of staining specificity). Only partial overlap with DEAD box helicase 5 (DDX5), a common component of various neuronal RNP granules and particles (12,33–36), was observed (Fig. 4F). We hence concluded that FGs represent a distinct type of RNA granule, which nevertheless sequester certain other RNA-binding proteins normally found in mammalian RNP particles. Predictably, FAs originated from FGs were also strongly positive for DDX5 (Supplementary Material, Fig. S5B). Although many components of neuronal RNA granules, such as FMRP, Y12 and staufen1, are recruited to SGs (27), we demonstrated that DDX5 is not a constituent of arsenite-induced SGs (Supplementary Material, Fig. S5A). As illustrated in Supplementary Material, Figure S5C, FUS-containing SGs formed following arsenite treatment in cells with low level and diffuse distribution of cytoplasmic FUS are also negative for DDX5. Therefore, DDX5 protein can be used as a marker to distinguish between FAs and SGs.

### FAs disrupt formation of stress granules and P-bodies

**Figure 5.**
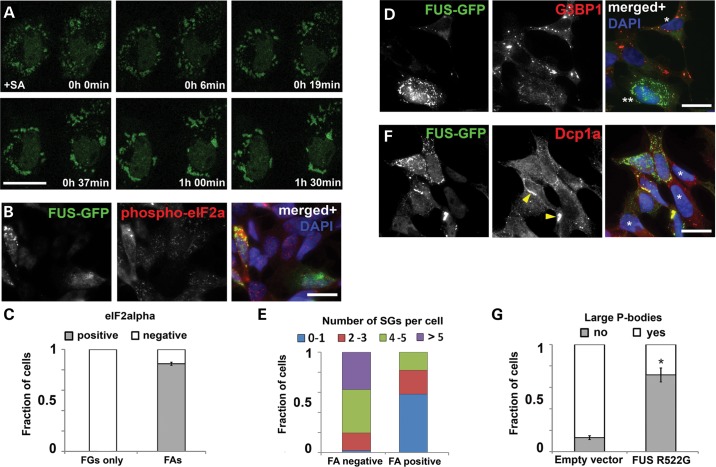
Formation of stress granules and P-bodies in SH-SY5Y cells is attenuated in the presence of FAs. (**A**) Time lapse confocal imaging reveals rapid, within 1.5 h, co-assembly of FGs into FAs in cells subjected to oxidative stress (sodium arsenite, SA). (**B** and **C**) The majority of cells with FAs display elevated levels phospho-eIF2alpha, a marker of activated cellular stress response, while levels of this protein are unaltered in cells bearing FGs only. (**D** and **E**) FUS R522G expressing cells with diffuse protein distribution form normally looking SGs upon stressful exposure (SA) as revealed by staining for a SG marker G3B1 (D, cell marked with one asterisk), while in cells with FUS accumulated in the form of FGs/FAs formation of SGs is impaired (D, cell marked with two asterisks, and quantified in E). (**F** and **G**) Cytoplasmically accumulated FUS prevents formation of P-bodies, and Dcp1a becomes sequestered by a subset of FAs. Cells were considered as having P-bodies if at least two large P-bodies were evident. Asterisks in (F) indicate non-transfected cells possessing multiple P-bodies and arrows point to FAs positive for Dcp1a. Bar chart shows means ± SEM, *P < 0.05. Scale bars, 10 µm.

Since RNA supply is critical for FAs formation and stability, stress conditions should favour FAs assembly and growth by providing transcripts running off ribosomes. Using confocal live imaging, we tracked the behaviour of preformed FGs in conditions of acute stress. Indeed, after addition of sodium arsenite, FGs started rapidly clustering together and assembling into FAs (Fig. 5A and Supplementary Material, Video S2). Therefore, clustering of FGs we detected in a fraction of naïve cells expressing cytoplasmic FUS (Fig. 1D and F) may indicate that a stress response has been triggered in these cells. As expected, in cells bearing only FGs the level of phosphorylated eIF2alpha protein, a reliable stress marker, was the same as in untransfected cells, but was markedly higher in the majority of cells with FAs (Fig. 5B,C, see also Supplementary Material, Fig. S4B for the control of staining specificity).

Based on the facts that FAs sequester proteins involved in SG nucleation and RNAs released from cellular translational machinery, one would expect them to interfere with SG formation under stress. To test this, we assessed the ability of cells with diffuse FUS distribution and those with FAs to generate SGs. Consistent with the previous reports, in the former cells typical SGs appeared in response to acute stress (sodium arsenite or heat shock) and they recruited FUS protein (examples shown in Supplementary Material, Fig. S6 and Video S3). These SGs were strongly TIAR/G3B1-positive and indistinguishable from SGs in non-transfected cells (Fig. 5D, cell marked with an asterisk). However, cells containing preformed FGs and/or FAs generally failed to form SGs or had fewer SGs per cell compared with cells with diffuse FUS distribution only (Fig. 5D and E).

Depletion of RNA by sequestration into FAs may also negatively affect P-bodies, sites of RNA degradation and triage. Indeed, P-bodies which are abundant in SH-SY5Y cells under basal conditions (Figs 1J and 5G) are lost in cells bearing FGs and/or aggregates: only a minor fraction of such cells contained more than two P-bodies per cell, and they generally were smaller in size and often completely absent. Moreover, some FAs were positive for Dcp1a protein (Fig. 5F, arrowheads). Therefore, FAs may disrupt P-body formation both via RNA depletion and by sequestration of their core components such as Dcp1a.

### Disruption of RNA-binding motifs of cytoplasmic FUS augments its aggregation

**Figure 6.**
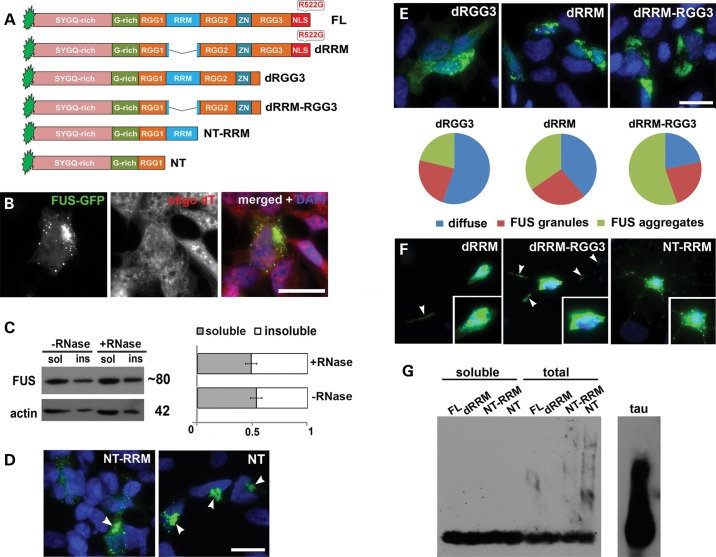
FUS with compromised structure of its RNA-binding moiety readily aggregates in the cytoplasm of SH-SY5Y cells. (**A**) Domain structure of proteins used to assess the contribution of RNA recognition and binding domains to FUS aggregation properties. (**B**) RNA-FISH with oligo(dT) probe demonstrates that neither small dot-like precursors of aggresomes nor aggresomes formed by FUS NT-RRM contain polyadenylated RNA. (**C**) Triton X-100 insoluble species of NT-RRM are insensitive to RNase A treatment. (**D**) 24 h after transfection, FUS NT is found almost exclusively in the form of aggresomes (arrowheads) while FUS NT-RRM forms aggresomes only in a fraction of cells. (**E**) Progressive disruption of RNA recognition and binding motifs of cytoplasmically localized FUS results in enhanced propensity to form FAs. FUS R522G with its RRM deleted (dRRM) is more frequently found in the form of FAs compared with dRGG variant; the fraction of cells with FAs increases further for a variant with both RRM and part of RGG3 domain deleted (dRGG3). (**F**) Aggregation patterns for RNA-binding deficient FUS variants detected in SH-SY5Y cells are recapitulated in primary hippocampal neurons. In addition to the cell body, accumulations of these FUS variants are often found in the processes (arrowheads). NT-RRM variant forms typical aggresomes alongside with multiple dot-like aggregates in the neurites. (**G**) SDD-AGE analysis shows SDS-sensitive, non-amyloid nature of FAs formed in cells in contrast to amyloid aggregates of tau protein in the spinal cord of transgenic mice (P301S line). Scale bars, 10 µm.

Results of the above experiments suggested that despite being clearly distinct from physiological RNP complexes, FGs and FAs are dynamic structures and not products of final irreversible aggregation of FUS that form inclusions typical for FUSopathies. The integrity of FAs formed by RNA-binding-competent FUS variants depends on the supply of non-polysomal RNA species. In contrast, a FUS variant deficient for RNA-binding readily and irreversibly aggregates with formation of emetine-resistant aggresome-like structures in cultured cells, and causes FUSopathy in transgenic mice (23,24). Here, we demonstrated that, as expected, neither small precursors, nor final aggresome-like aggregates formed by this variant (NT-RRM, Fig. 6A) in SH-SY5Y cells contain polyadenylated RNA (Fig. 6B) and that both types of structures are resistant to RNase treatment (Fig. 6C). A FUS variant with a longer deletion that includes the RRM domain (NT) formed similar juxtanuclear aggresomes (Fig. 6D) even faster because 24 h after transfection virtually all cells expressing NT protein possessed these structures and precursors could hardly be detected, whereas aggresomes are observed only in ∼35% of cells expressing NT-RRM (23). Typical aggresomes were also formed by NT-RRM and NT variants in primary neurons (Fig. 6F, right panel and data not shown). Therefore, it was feasible to further investigate to what extent RNA-binding ability regulates FUS aggregation *in vivo*. To address this, GFP-fusion FUS variants with different combinations of RNA-binding domain deletions were expressed in neuroblastoma cells (Fig. 6A). Deletion of only a portion of FUS RGG3 motif in dRGG3 protein does not affect frequency of cells with different types of the protein distribution in the cytoplasm compared with FUS variants with compromised NLS but intact RNA-binding domains, e.g. FUS R522G (compare Fig. 1F and Fig. 6E). Upon deletion of RRM (dRRM variant), the ratio of cells with FAs to cells with diffuse FUS distribution increased and when RRM deletion was accompanied by deletion of a portion of RGG3 motif (dRRM-RGG3 variant), cells with FAs became a predominant class (Fig. 6E). Aggregation patterns detected for dRRM and dRRM-RGG3 in neuroblastoma cells were recapitulated in primary neurons (Fig. 6F, left and middle panels). Interestingly, in contrast to full-length cytoplasmic FUS, FGs formed by these variants often accumulated in neuronal processes, a finding possibly also reflecting their higher aggregation propensity (Fig. 6F, arrowheads). However, all of these deletions do not affect the ability of proteins to form FGs as proportion of cells bearing these structures only was approximately the same for these variants and RNA-binding competent FUS variants. Taken together, these results suggested а direct correlation between the degree of RNA-binding impairment and the propensity to form cytoplasmic FAs. Furthermore, they show that FUS with significantly compromised RNA binding acquires the ability to aggregate in a principally different way, not involving formation of RNA-containing FGs or FAs. Nonetheless, aggregates formed by all FUS variants were SDS-sensitive when run on SDD-AGE indicating their non-amyloid nature (Fig. 6G).

### Inhibition of transcription triggers RNA-independent aggregation of RNA-binding competent FUS in cell cytoplasm

**Figure 7.**
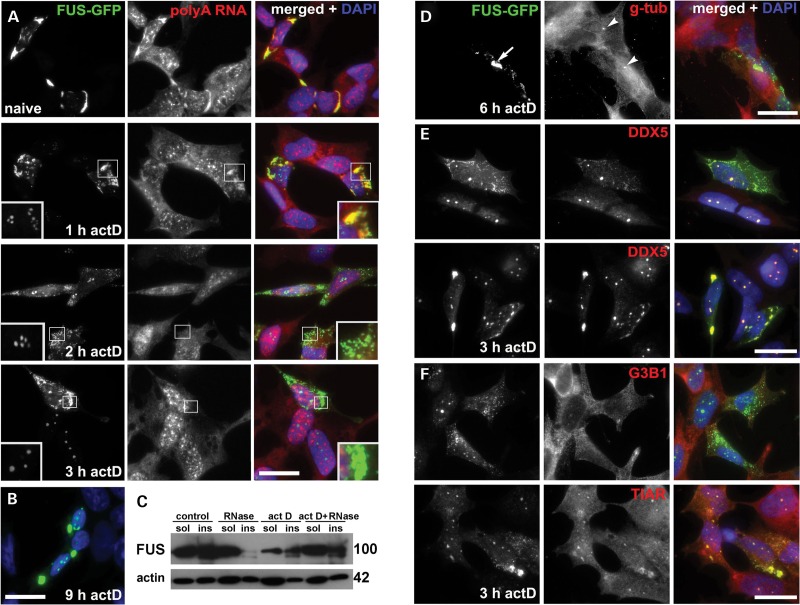
Dissipation of FAs and FGs and subsequent RNA-independent FUS aggregation following inhibition of transcription in SH-SY5Y and COS7 cells expressing cytoplasmically localized FUS R522G. (**A**) RNA-FISH with oligo(dT) probe shows dynamics of polyadenylated RNA loss from FAs and FGs and secondary aggregation leading to the formation of RNA-independent FAs (FA(-)s). Inserts in the left bottom corner represent enlarged nucleolar caps from a representative cell in this image; channel intensity in insets is enhanced where appropriate. Note changes in the morphology of FUS-positive nucleolar caps reflecting the duration of transcriptional inhibition: several crescent-shaped caps after 1–2 h of actinomycin D treatment replaced by dot-like FUS accumulation in their fibrillar center from 3 h onwards. (**B**) Products of secondary RNA-independent aggregation fuse to each other to produce large aggregates after prolonged (9 h) exposure to actinomycin D. (**C**) Triton X-100-insoluble FUS species recovered from COS7 cells after 6 h actinomycin D treatment are insensitive to RNase A. (**D**) Large FA(-)s (arrow) formed after prolonged (for 6 h) transcriptional repression in SH-SY5Y cells are found in the immediate vicinity of the centrosome (arrowheads) visualized by anti-γ-tubulin (g-tub) staining. DDX5 but not G3B1 co-aggregates with FUS R522G in small (**E**) and large (**F**) FA(-)s detected after 3 h of transcriptional inhibition with actinomycin D (act D). Scale bar (A, B, D, E) 10 µm; (F) 2.5 µm.

To recapitulate attenuated protein–RNA interaction for full-length RNA-binding competent FUS isoforms, we reduced supply of newly synthesized transcripts using transcriptional inhibition by actinomycin D. Using RNA-FISH with oligo(dT) probe, we demonstrated that treatment with actinomycin D at a concentration inhibiting all three RNA polymerases causes not only fast dissipation of FAs formed by R522G FUS but also nearly complete depletion of polyadenylated RNA from all small FUS-positive granules preserved in the cell cytoplasm after 2 h treatment, i.e. from products of FAs decay and preexisting FGs (Fig. 7A). Unexpectedly, longer actinomycin D treatment led to fusing and clustering of these granules and re-appearance of large FAs in virtually all cells with high level of R522G FUS expression (Fig. 7A and B). A similar pattern was observed in COS7 cells (Supplementary Material, Fig. S7C), in neuroblastoma cells expressing dRGG3 and FUS 1–513, in cells transfected with Flag-tagged R522G FUS (Supplementary Material, Fig. S7B) and upon treatment with another transcriptional inhibitor selectively targeting RNA polymerase II, DRB (Supplementary Material, Fig. S7A). However, these aggregates were different from FAs as they remained RNA-negative and resistant to RNase treatment (Fig. 7A and C). Therefore, a designation FA(-)s was chosen for aggregates formed by RNA-binding competent FUS following transcriptional arrest. At the final stage of their genesis, FA(-)s formed juxtanuclear structures in close vicinity to the centrosome (Fig. 7D), similarly to aggresomes formed by NT-RRM FUS variant (23). But unlike aggresomes and their precursors in cells expressing RNA-binding deficient FUS variants, FA(-)s were positive to DDX5 (compare Fig. 7E and Supplementary Material, Fig. S8), which is consistent with their origin from FAs and FGs that are also positive for this protein (Fig. 4G and Supplementary Material, Fig. S5B). In contrast, FA(-)s were always negative for G3B1 and only weakly positive for TIAR (Fig. 7F), proteins often found in FAs (Fig. 1H) indicating selective loss of proteins during dissociation of FAs and selective co-aggregation during secondary, RNA-independent FUS aggregation.

### Transcriptional inhibition results in pathological redistribution and aggregation of predominantly nuclear FUS mutants

**Figure 8.**
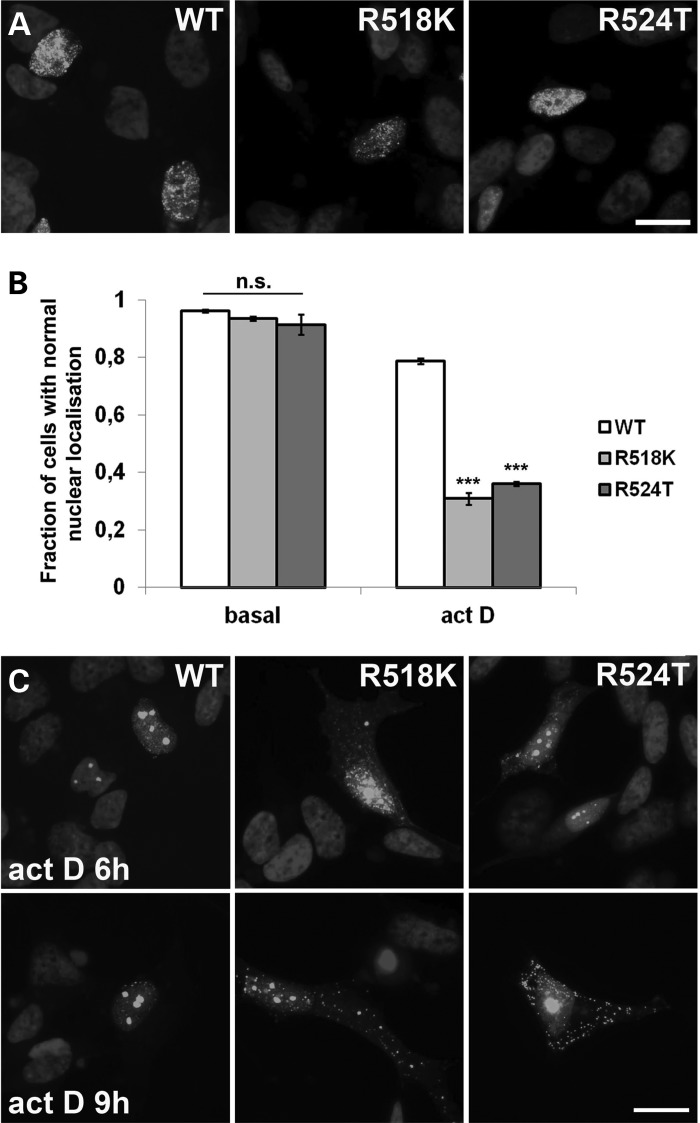
Redistribution of FUS and its mutant variants to the cytoplasm of SH-SY5Y cells following transcriptional arrest. (**A** and **B**) Like wild-type (WT) FUS, its variants bearing substitutions R524T or R518K in the NLS display nuclear localization in the majority of cells (A). Treatment with actinomycin D for 2 h dramatically increases the number of cells with FUS R524T and FUS R518K partially shifted to the cytoplasm while the distribution of wild-type protein is much less affected (B). (**C**) Prolonged, for 6 or 9 h, treatment with actinomycin D causes formation of cytoplasmic aggregates by FUS R524T and FUS R518K variants. Bar chart in (B) shows means ± SEM, ***P < 0.005. Scale bars, 10 µm.

Significant shift of FUS from the nucleus to the cytoplasm of motor neurons is an important event in the pathogenesis of ALS-FUS. A number of ALS-associated FUS mutations affecting NLS (e.g. R522G and R525L) considerably impair nuclear transport leading to predominantly cytoplasmic localization of FUS in cultured cells; however, certain other mutations in this region do not cause such a redistribution. For example, R518K and R524T mutants display nuclear localization indistinguishable from that of wild-type protein in neuroblastoma cell line (Fig. 8A). It has been previously demonstrated that endogenous FUS and other FET family members undergo a partial shift to cytoplasm following inhibition of transcription (37,38). To assess effects of transcriptional arrest on FUS variants bearing a mutation in NLS but retaining nuclear localization, we treated cells expressing wild-type, R518K or R524T variants of FUS with actinomycin D. After 2 h in the majority of cells expressing either mutant, a significant portion of the protein was shifted to the cytoplasm (Fig. 8B). Similar shift was observed in less than 20% of cells expressing wild-type FUS. Longer treatments (6 and 9 h) resulted not only in redistribution, but also in progressive aggregation of mutant FUS in the cytoplasm (Fig. 8C). The wild-type protein also gradually accumulated in the cytoplasm (Fig. 8) although not to the same extent as mutant variants. Therefore, transcriptional inhibition may not only become a trigger for RNA-independent aggregation of FUS but also facilitate FUS accumulation in the cytoplasm, and this process is more efficient when NLS is impaired.

## DISCUSSION

A common trait of several neurodegenerative diseases, including ALS and FTLD, is the presence in the patients' nervous system of intracellular inclusions representing final products of pathological aggregation of certain RNA-binding proteins (reviewed in 39). These proteins typically possess prion-like, also known as low-complexity, domains required for their reversible aggregation, which is an important process in biogenesis of physiological RNP granules (30,40). It has been shown that inclusions formed by TDP-43 and FUS, RNA-binding proteins etiologically and pathogenically involved in ALS and FTLD, contain protein constituents of normal RNP complexes (15,41,42). Together, these observations led to a seemingly logical notion that inclusions are products of pathological transformation of certain physiologically relevant RNP structures such as SGs (15,43,44). However, the ability to bind target RNA, which is a prerequisite for physiological aggregation of TDP-43, FUS and related proteins (45,46), is not compulsory for their pathological aggregation as illustrated by our recent studies of FUS in cells and transgenic mice (23,24). Moreover, our data suggested that compromised ability to be sequestered into SGs due to deletions of major RNA-binding domains augments aggregation of FUS mislocalized in the cytoplasm (23). This raises several questions. Is the mechanism of inclusion formation by RNA-binding-incompetent FUS completely different from the mechanism involving FUS variants capable of RNA binding and entering physiological RNA granules? What are the differences and similarities between FUS-containing RNA granules in unaffected cells and in cells accumulating large amount of FUS in their cytoplasm? How do the conditions of RNA deficiency affect aggregation of FUS? Answers to these questions might become crucial for choosing between molecules and processes that could be or should not be considered as potential therapeutic targets for FUSopathies and potentially to other diseases that can be described as RNPopathies.

**Figure 9.**
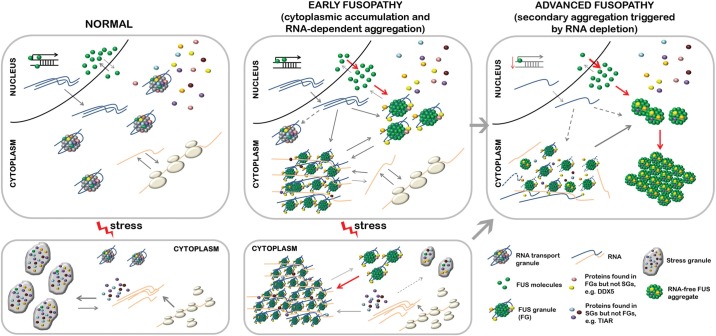
A model of FUS aggregation in cultured cells and its extrapolation to human FUSopathies. (Left panels) In a normal cell unaffected by FUSopathy, FUS resides predominantly in the nucleus, and cytoplasmic pool of the protein contributes to the formation of physiological RNA transport granules. When the cell is subjected to an external stress, translation of certain transcripts is blocked, they become released from polysomes and sequestered into stress granules. RNA transport granules and stress granules share a specific pool of proteins. (Middle panels) In cells with significant shift of FUS from the nucleus to the cytoplasm (due to a mutation, posttranslational modification(s) or transportin defect) accumulated protein nucleates pathological RNA granules, FGs. This happens after certain concentration threshold of cytoplasmic FUS is reached and does not require exposure to external stress factors. FGs recruit newly synthesized transcripts and certain proteins, for example, those present in RNA transport granules. FGs cluster, and recruit further non-polysomal transcripts as well as specific proteins including typical stress granule components, finally forming higher-order structures, FAs. This clustering is enhanced by endogenous or exogenous stress that increases the availability of free RNA. The presence of FAs that sequester SG proteins and RNAs, interferes with SG (and P-bodies, nor shown in the scheme) formation in affected cells. These processes occur at the early stage of FUSopathy development, when the cell does not contain FUS inclusions, which are histopathological hallmarks of the disease. (Right panels) Progressive clearance of FUS from the nucleus leads to alterations in transcription, which can be exacerbated by ageing. Reduced transcription rates and therefore availability of cognate RNAs results in the collapse of FAs. Products of FAs dissolution undergo RNA-independent aggregation and deposit in non-RNA based aggregates. This stage corresponds to the advanced stage of FUSopathy development characterized by severe cellular dysfunction and formation of pathological FUS inclusions.

Therefore, we carried out detailed analysis of RNP granules formed in cultured cells following aberrant cytoplasmic accumulation of FUS. We found that redistribution of diffuse cytoplasmic FUS to granule-like structures correlates with increased accumulation of the protein, suggesting that this morphological change is triggered after a certain concentration threshold has been reached. The first products of this process are small round particles, FGs, which can later cluster together, sequester further RNA and protein molecules, and form larger structures, FAs (illustrated in Fig. 9, middle top panel). The presence of a prion-like domain at the N-terminus of FUS is required for formation of FGs, but their integrity also depends on the interaction of FUS with specific RNAs because RNase treatment dissociates these detergent-resistant complexes. Moreover, RNA-binding domains of proteins that have an overlapping RNA-interactome with FUS can substitute its RNA-binding domains for FGs formation. These results once again emphasize the important role of prion-like domain (47–50). They are also consistent with recently described role of RNA in seeding higher-order assembly of FUS *in vitro* (51). Thus, the two-stage model previously proposed for RNA particles (40) can also be applied to describe formation of FGs. According to this model, an RNA-binding protein first binds specific RNA(s) and only then its prion-like domain facilitates phase transition from soluble to reversibly aggregated state.

FGs resemble RNA transport granules, RNA–protein entities particularly abundant in neurons and comprising factors that are necessary for temporary translational silencing of mRNAs, their large distance transport from soma to processes and local translation, such as FMRP, staufen1 and Puralpha (52–55). FUS was previously identified as an auxiliary component of these physiological RNA granules (12,31). However, no co-localization of cytoplasmic FUS and staufen1 has been found (21), and we demonstrated that FGs are completely negative for another major constituent of RNA granules, FMRP and only a fraction of FGs are positive for DDX5. The presence in neuronal RNA transport granules of stalled translational complexes is well established (18,56–58), but we did not detect S6 ribosomal protein in FGs. This strongly suggests that despite certain structural similarities, FGs are distinct from physiological RNA transport granules.

Like FUS, a related RNA-binding protein TDP-43 is a component of neuronal RNA transport granules (35,59,60), but in our studies this protein was unable to nucleate RNA granules that were similar to FGs, even when accumulated in the cytoplasm at significant levels. This difference in the behaviour of the two proteins may be down to the ‘strength’ of their prion-like domains. While FUS belongs to the top 15 proteins based on the similarity of its predicted prion-like domain to yeast prion domains, TDP-43 is only 63rd on this list (28,61). A potent prion-like domain thus might be a critical factor defining the ability to form RNA granules independent of core RNA transport granule proteins such as staufens or FMRP. High propensity of cytoplasmically mislocalized FUS to nucleate FGs might contribute to the early stages of neuronal pathology development in FUSopathies by sequestration in the neuron cell body of a significant pool of mRNAs that normally should have been transported to neurites and synaptic terminals by RNA transport granules. This might lead to inadequate local protein production and consequent progressive synaptic dysfunction.

Cellular stress, caused by external factors, led to clustering of FGs with formation of FAs (illustrated in Fig. 9, middle bottom panel). Moreover, in the absence of external stress, the presence of FAs correlated with a substantially increased level in the same cells of phospho-eIF2alpha, a marker of activated cellular stress response, suggesting a possible role in FAs formation of internal stress factors, most probably pathological accumulation of FUS itself. Formation of FAs is promoted by increased availability of polyadenylated RNA released from polysomes in stressed cells and requires specific interaction of FUS with a pool of target RNAs within this fraction. These features along with sharing some protein components make FAs similar to SGs. However, high resolution and time lapse imaging revealed that FAs represent agglomerations of FGs that are prone to further fusion with each other, while mature SGs are structurally uniform and rarely fuse. Moreover, FAs retain markers characteristic for their precursors, FGs, that are not present in SGs, for example DDX5. Dcp1a, a core P-body component, is also often detected in FAs, while SGs are free of this protein (62). On the other hand, FAs are negative for ribosomal protein S6, suggesting that in contrast to SGs stalled translational complexes are not their major component. Although some of FAs are positive for TIAR, this typical SG marker readily dissociates from FAs following RNase digestion of cell extracts or mildly fixed cells, while this treatment has no effect on TIAR associated with SGs. These results clearly demonstrated that FAs and SGs are two distinct types of RNA granules. However, FAs can hijack normal constituents of SGs and other cytoplasmic RNP complexes, i.e. RNA species and RNA-binding proteins, disrupting normal intracellular RNA metabolism and stress responses. Indeed, the number of P-bodies is diminished and the ability to form SGs is compromised in cells already bearing FAs. FUS folding in FGs and FAs is likely to be normal, i.e. similar to that of FUS in transport granules. It is tempting to speculate that this can deceive cellular systems responsible for clearance of abnormally folded and pathologically aggregated proteins allowing significant accumulation of pseudophysiological FUS-containing structures in the cytoplasm.

FAs are dynamic structures and their stability depends on the presence of cognate RNAs because when the supply of these RNAs is reduced by pharmacological inhibition of mRNA synthesis or stalling mRNA in pre-initiation complexes, FAs rapidly lose their integral RNA and dissociate into smaller RNA-free granules. In conditions of continuing transcriptional arrest, granules become substrates of RNA-independent fusion (illustrated in Fig. 9, right top panel). In cultured cells, the final products of this fusion are large juxtanuclear aggresomes similar to those formed by FUS variants unable to bind RNA due to the loss of functional RNA-binding domains.

FUS plays an important role in regulation of transcription (63–68) and disruption of this process due to the nuclear depletion of FUS might contribute to attenuated transcription common to human FUSopathies. Furthermore, reduced availability of newly synthesized transcripts can initiate RNA-independent FUS aggregation. Ageing is another condition known to be associated with decreased transcriptional rate (reviewed in 69) and thus likely to contribute to progression of certain FUSopathies.

Recently, we have demonstrated that neuronal expression of FUS variants capable to form RNA-free aggregates causes severe neurological phenotype in transgenic mice (23,24) accompanied by formation of multiple intraneuronal inclusions. This suggests that FAs structurally similar to RNA-free FA(-)s described here for a model cell culture system might be ultimately responsible for the development of human FUSopathies.

In conclusion, our study provided evidence that a multistep process of FUS aggregation in the cell cytoplasm includes RNA-dependent and RNA-independent mechanisms. A similar chain of events might be involved in formation of intracellular inclusions by FUS and some other RNA-binding proteins implicated in pathogenesis of human RNPopathies.

## MATERIALS AND METHODS

### Expression plasmids

Full-length FUS, TDP-43 lacking its NLS, FUS fragments carrying mutations, fragments of TDP-43, Npl3 and Sup35 proteins were produced by PCR amplification from full-length cDNA using corresponding primers, cloned into pTOPO-Blunt vector (Invitrogen) and subcloned into the pEGFP-C1 vector (Clontech) or pFLAG-CMV-4 vector (Sigma). In FUS-Npl3 chimera, major RNA-binding domains of FUS (amino acids 360–526) were replaced by RRMs (yRRM1 and yRRM2) of the yeast RNA-binding protein Npl3. In FUS-TDP-43 chimera, the same FUS domains were substituted by RRM1 and RRM2 of TDP-43 protein. In Sup-35-FUS chimera, N-terminal part (amino acids 1–359) of FUS comprising the prion-like domain was replaced by the prion domain of the yeast protein Sup35 (amino acids1–125).

### Cell lines, transfection and treatments

SH-SY5Y human neuroblastoma, COS7, MCF7 and HEK293 cells were maintained in Dulbecco modified Eagle medium (Invitrogen), supplemented with 10% fetal bovine serum. For immunofluorescence, cells were grown on poly-l-lysine coated cover slips as described elsewhere (23,70). Cells were transfected with expression plasmids using Lipofectamine2000 (Invitrogen) according to the manufacturer's instructions. For oxidative stress induction, cells were exposed to 0.5 mm sodium arsenite (Sigma) for 1 h. Cells were treated with 20 µg/ml emetine (Sigma) to inhibit translation, and with 5 µg/ml actinomycin D (Calbiochem) or 25 μg/ml 5,6-dicholoro-β-d-ribofuranosylbenzimidazole (DRB, Sigma) to inhibit transcription. Changes in the morphology of FUS-positive nucleolar caps in cells from the same view field were used as indicators of the efficiency and duration of transcriptional inhibition: several crescent-shaped caps after 1–2 h of actinomycin D treatment replaced by dot-like FUS accumulation in their fibrillar centre from 3 h onwards.

#### Primary mouse hippocampal cultures

All reagents used for preparation of hippocampal cultures were purchased from Invitrogen unless stated otherwise. Hippocampi were dissected from mice at postnatal day 3, digested for 40 min in 0.1% trypsin in HBSS supplemented with 10 mm Hepes and 1 mm pyruvate. After mechanical dissociation in Neurobasal A medium containing 50 U/ml penicillin/streptomycin, 0.2% β-mercaptoethanol (Sigma), 500 µm l-glutamine and 10% horse serum hippocampi were centrifuged for 5 min at 1500 rpm. Pellets were resuspended in fresh medium and plated on poly-l-lysine coated cover slips. One day after plating, the medium was changed to serum-free medium containing B27. Mixed neuronal-glial cultures were transfected on DIV7 using Lipofectamine2000 according to the standard procedure except lipofectamine–DNA complexes were left for 1 h and subsequently replaced with normal culture medium. Cells were fixed and stained 48 h after transfection.

### Immunofluorescence

Cells on cover slips were fixed with 4% paraformaldehyde on ice for 15 min, followed by washes with PBS and 5 min permebealization in cold methanol. Blocking in 5% goat serum/PBS/0.1% Triton X-100, incubation with primary (see below) and secondary Alexa Fluor-conjugated anti-mouse or anti-rabbit immunoglobulins (1:1000; Molecular Probes, Invitrogen), washes and DAPI counterstaining were performed as previously (70). RNase digestion on cover slips was performed as described in ref. (71). Fluorescent images were taken either using BX61 microscope (Olympus; UPlanFI 100×/1.30 oil objective), F-View II camera (Olympus) and CellF software; or Leica TCS SP2 MP confocal microscope (Leica Microsystems, see *Live cell imaging* section). Confocal images were processed using Leica Confocal Software (Leica Microsystems). Fluorescence intensity was measured 24 h after transfection with plasmids encoding GFP-fusion proteins in three non-overlapping 2.5 × 2.5 µm squares randomly (regardless of the presence or absence of FGs in this area) chosen in the cytoplasm of a GFP-positive cell using free-access ImageJ software and mean intensity for each cell was calculated. For all quantifications, at least 150 cells were counted from three independent experiments unless otherwise indicated.

### RNA fluorescent *in situ* hybridization

A previously described protocol (72) modified for fluorescently labelled oligo(dT) probe was used. Briefly, paraformaldehyde-fixed and permeabilized cells were incubated in 70% ethanol for 10 min followed by 1 m Tris pH 8.0 for 5 min. To detect poly(A)+ mRNA, cells were incubated at 37°C overnight with 1 μm Cy5-labelled oligo(dT)30 probe diluted in hybridization buffer (2× SSC, 25% formamide, 10% dextran sulphate, 0.005% BSA, 1 mg/ml yeast tRNA). After washes, cells were incubated with antibodies against FUS (C-terminus specific, mouse monoclonal, Santa Cruz Biotechnology), diluted in 2× SSC/ 0.1% Triton X-100 for 3 h at room temperature, followed by incubation with Alexa Fluor 488-conjugated secondary antibodies and DAPI. For RNA-FISH and immunofluorescence applications, images were taken using BX61 microscope (Olympus) and processed using CellF software (Olympus).

### Live cell imaging

Time-lapse images were obtained using a Leica TCS SP2 MP confocal microscope, equipped with an on-scope incubator with temperature control (Leica Microsystems). COS7 cells were plated on glass-bottomed tissue culture dishes and transfected with FUS R522G construct as described above. Twenty four hours post-transfection, regular culture media was replaced with HEPES-buffered media (10 mm HEPES-KOH, pH7.5) for maintaining physiological pH outside a CO2 incubator, and where appropriate sodium arsenite was added to cells at final concentration of 0.5 mm. Cells were visualized under Fluotar L 63 × 1.4 oil objective. A sequence of images taken with 5 or 8 min intervals was further transformed into a movie using Leica Application Suite AF software.

### Antibodies

Commercially available primary antibodies against the following antigens were used: GFP (mouse monoclonal, clone 3A9, Protein Synthesis and Living Colours, Clontech); FUS (mouse monoclonal against C-terminus, Santa Cruz Biotechnology and rabbit polyclonal against N-terminus, Abcam); TIAR (mouse monoclonal, clone 6, BD Biosciences); G3BP1 (mouse monoclonal, clone 23/G3BP, BD Biosciences); DDX5 (rabbit monoclonal, clone D15E10, Cell Signaling); Dcp1a (rabbit polyclonal against C-terminus, Sigma); FMRP (rabbit polyclonal, clone F4055, Sigma); phospho-eIF2alpha (rabbit polyclonal, Abcam); S6 Ribosomal Protein (rabbit polyclonal; Cell Signaling); beta-actin (mouse polyclonal, Sigma); GAPDH (mouse monoclonal; Santa Cruz Biotechnology). All primary antibodies were used in 1:1000 dilution for all applications.

### Fractionation into soluble/insoluble and granule-enriched fractions

To obtain detergent soluble and insoluble (aggregated) fractions, cells were scraped on ice in lysis buffer (PBS containing 0.1% Triton X-100 and protease inhibitors cocktail (Roche)) and left on ice for 30 min with periodic vortexing. Cell lysates were centrifuged at 17 000 g for 20 min at 4°C and supernatant and pellet were recovered as soluble and insoluble fractions, respectively. In case of RNase A treatment, the enzyme was added to lysates to yield final concentration of 1 mg/ml, and the lysates were left at room temperature for 25 min followed by centrifugation as described. For isolation of granule-enriched fraction, after lysis cell nuclei were removed by centrifugation at 1000 g for 10 min and large organelles and aggregated species—by centrifugation at 17 000 g for 20 min at 4°C. Resulting supernatant was further subjected to high-speed centrifugation (100 000 g for 20 min at 4°C) to give granule-enriched fraction (pellet, P100k) and soluble proteins (supernatant, S100k). Equal proportions of soluble and aggregated fractions were analysed on western blots.

### Western blotting

Protein sample preparation, SDS–PAGE, electroblotting, incubation with antibodies and ECL detection were carried out as described previously (73). Equal loading was confirmed by re-probing membranes with antibodies against beta-actin. Protein levels in soluble and insoluble fractions were measured using Alpha Innotech software, and a mean from three independent experiments was used for the final graph.

### SDD-AGE

Previously described protocol (74) was used with minor modifications. Briefly, cells were harvested 24 h post-transfection in lysis buffer, left on ice for 20 min with periodic vortexing and centrifuged at 1000 g to remove nuclei. Half of the resulting supernatant was kept as total cytoplasmic protein and the second half was centrifuged at 17 000 g and the supernatant was recovered as soluble cytoplasmic protein. To obtain a positive control for the presence of amyloid-type aggregates, the spinal cord of a 6-month-old transgenic TauP301S mouse was homogenized in lysis buffer and centrifuged at 17 000 g, the supernatant was used for SDD-AGE. Samples were mixed with equal amounts of 2X SDD-AGE loading buffer (1X TAE, 5% glycerol and 1% SDS) and run in 1.5% agarose containing 0.1% SDS. Proteins from the gel were transferred to nitrocellulose membrane using capillary transfer and the membrane was subjected to western blotting as described above using rabbit polyclonal anti-FUS antibody.

### Statistics

Statistical analysis was performed with the Mann–Whitney *U*-test using IBM SPSS Statistics software (IBM).

## SUPPLEMENTARY MATERIAL

Supplementary Material is available at *HMG* online.

## FUNDING

This work was supported by grants from The Welcome Trust (075615/Z/04/z) and Motor Neurone Disease Association (Buchman/Apr13/6096) to V.L.B., and grants from the Russian Foundation for Basic Research to TAS (RFBR N14-04-00796) and NN (RFBR 13-04-01633 A). H.R. was supported by the Cardiff NMHRI 4-year PhD Studentship Programme. Funding to pay the Open Access publication charges for this article was provided by The Wellcome Trust.

## Supplementary Material

Supplementary Data
