# Supplementary material for: Multistep process of FUS aggregation in the cell cytoplasm involves RNA-dependent and RNA-independent mechanisms
Source: Hum Mol Genet. 2014 May 19;23(19):5211–26. doi: 10.1093/hmg/ddu243 (PMC4159159; doi:10.1093/hmg/ddu243)
Supplement: Supplementary Data [file supp_23_19_5211__index.html]

Multistep process of FUS aggregation in the cell cytoplasm involves RNA-dependent and RNA-independent mechanisms — Multistep process of FUS aggregation in the cell cytoplasm involves RNA-dependent and RNA-independent mechanisms — Supplementary Data 

# Multistep process of FUS aggregation in the cell cytoplasm involves RNA-dependent and RNA-independent mechanisms

## Supplementary Data

Supplementary Data

**Files in this Data Supplement:**

- Supplementary Data - Docx file
- Supplementary Video 1 - mov file
- Supplementary Video 2 - mov file
- Supplementary Video 3 - mov file
