# Supplementary material for: Multistep process of FUS aggregation in the cell cytoplasm involves RNA-dependent and RNA-independent mechanisms
Source: Hum Mol Genet. 2014 May 19;23(19):5211–26. doi: 10.1093/hmg/ddu243 (PMC4159159; doi:10.1093/hmg/ddu243)
Supplement: Supplementary Data [file supp_ddu243_ddu243supp.docx]

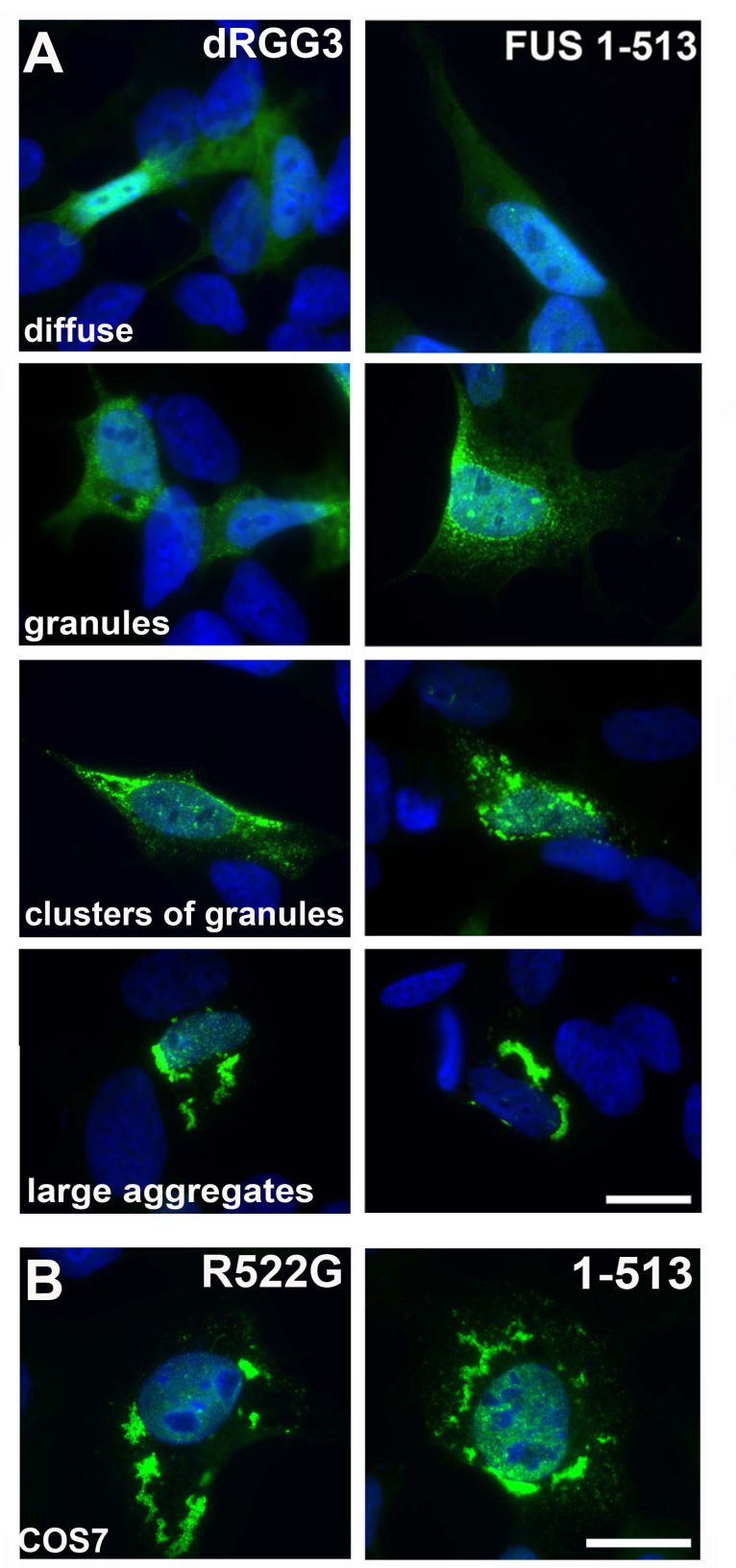


**Figure S1. Aggregation patterns of cytoplasmically localised GFP-tagged FUS variants in SH-SY5Y and COS7 cells.** (A) FUS lacking 60 (NLS and a portion of RGG3, dRGG3) or 13 amino acids (NLS) at its C-terminus can be either diffusely distributed or form granules, their clusters and large aggregates in SH-SY5Y cells, similar to the cytoplasmic variant bearing R522G substitution. The frequency of each of these types in a population of cells expressing this variant is comparable to their frequency in a population of cells expressing GFP-FUS R522G variant, compare Figure 1F and Figure 6E. (B) FUS R522G and FUS 1-513 form cytoplasmic aggregates in COS7 cells, which are morphologically similar to aggregates formed in SH-SY5Y cells. Scale bar, 10 µm.


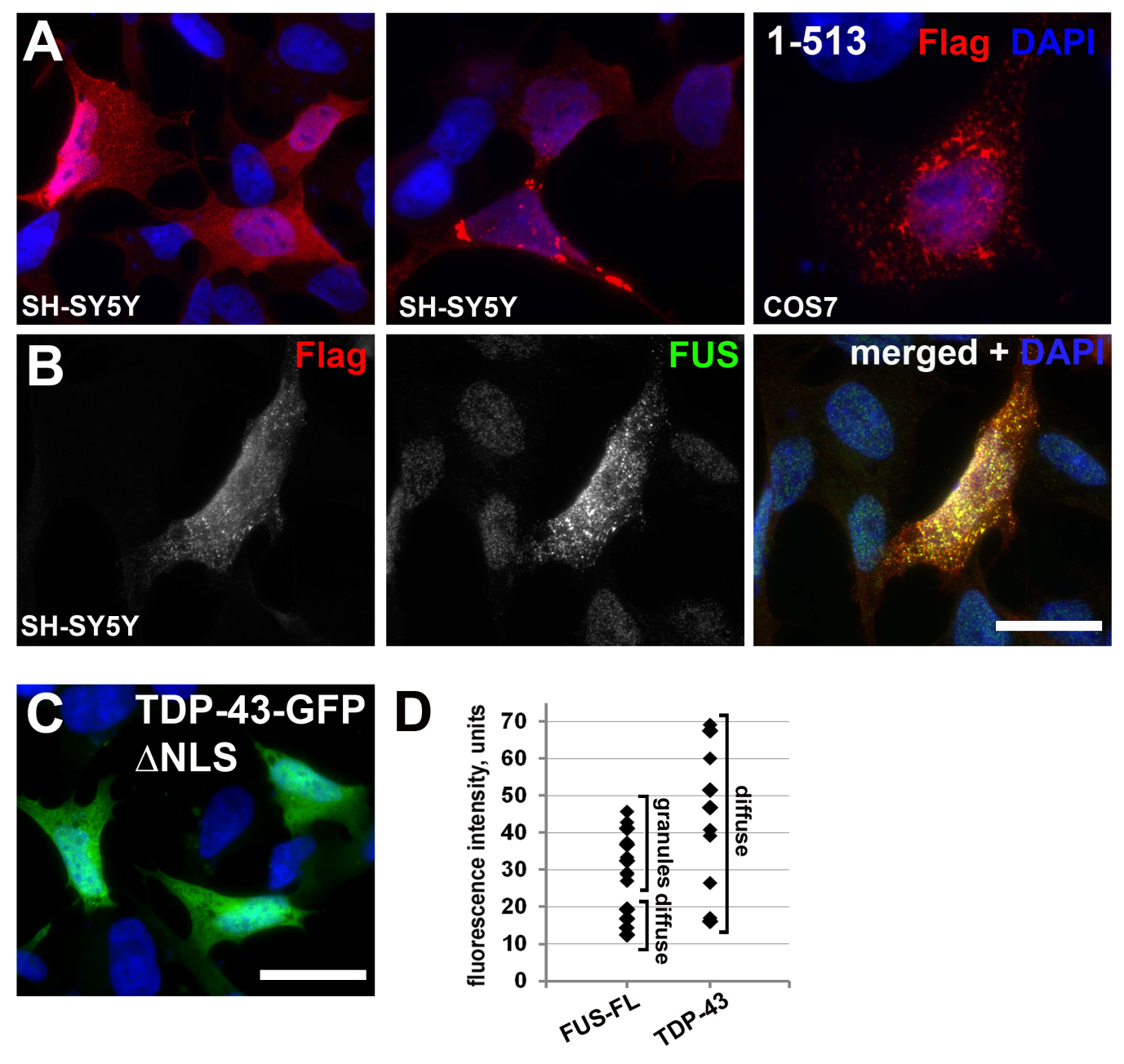


**Figure S2. Flag-tagged variants behave similarly to GFP-tagged FUS while mislocalized TDP-43 does not form aggregates in the cell cytoplasm.** (A, B) Flag-tagged FUS 1-513 is either diffusely distributed or forms granules/aggregates in SH-SY5Y and COS7 cells. In B double staining with anti-Flag and anti-FUS antibodies is shown. (C,D) TDP-43 with deleted NLS is diffusely distributed in the cytoplasm of SH-SY5Y cells even in cells with high level of expression (fluorescence intensity values for 12 individual cells were plotted). Scale bars, 15 µm.

**
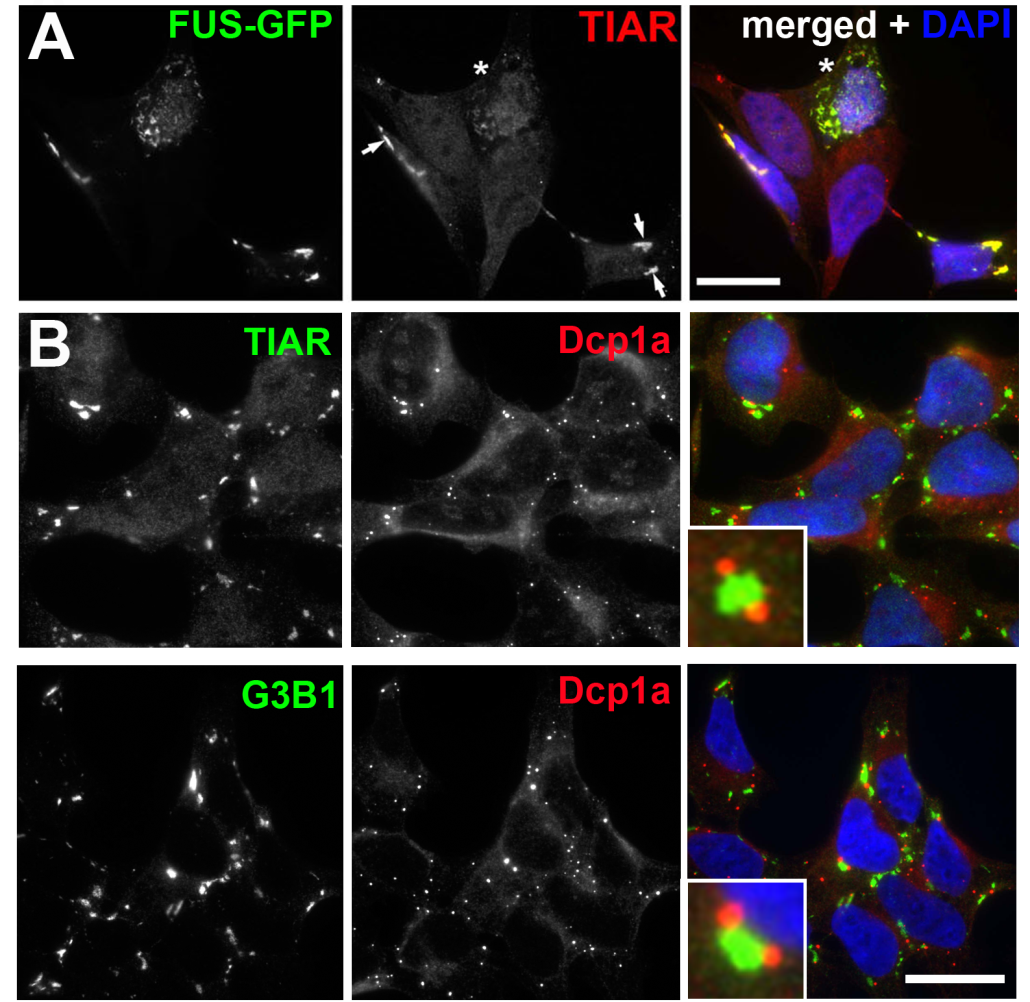
**

**Figure S3. FAs, SGs and P-bodies in neuroblastoma SH-SY5Y cells.** (A) Although some FAs are strongly positive for a SG marker TIAR (arrows), a fraction of FAs displays only weak TIAR immunoreactivity (cell marked with an asterisk). (B) P-bodies revealed by immunostaining with anti-Dcp1a antibody in naïve cells are found in the immediate vicinity of SGs visualized with anti-TIAR or G3B1 antibodies but these RNP structures do not overlap. Scale bar, 10 µm.

**
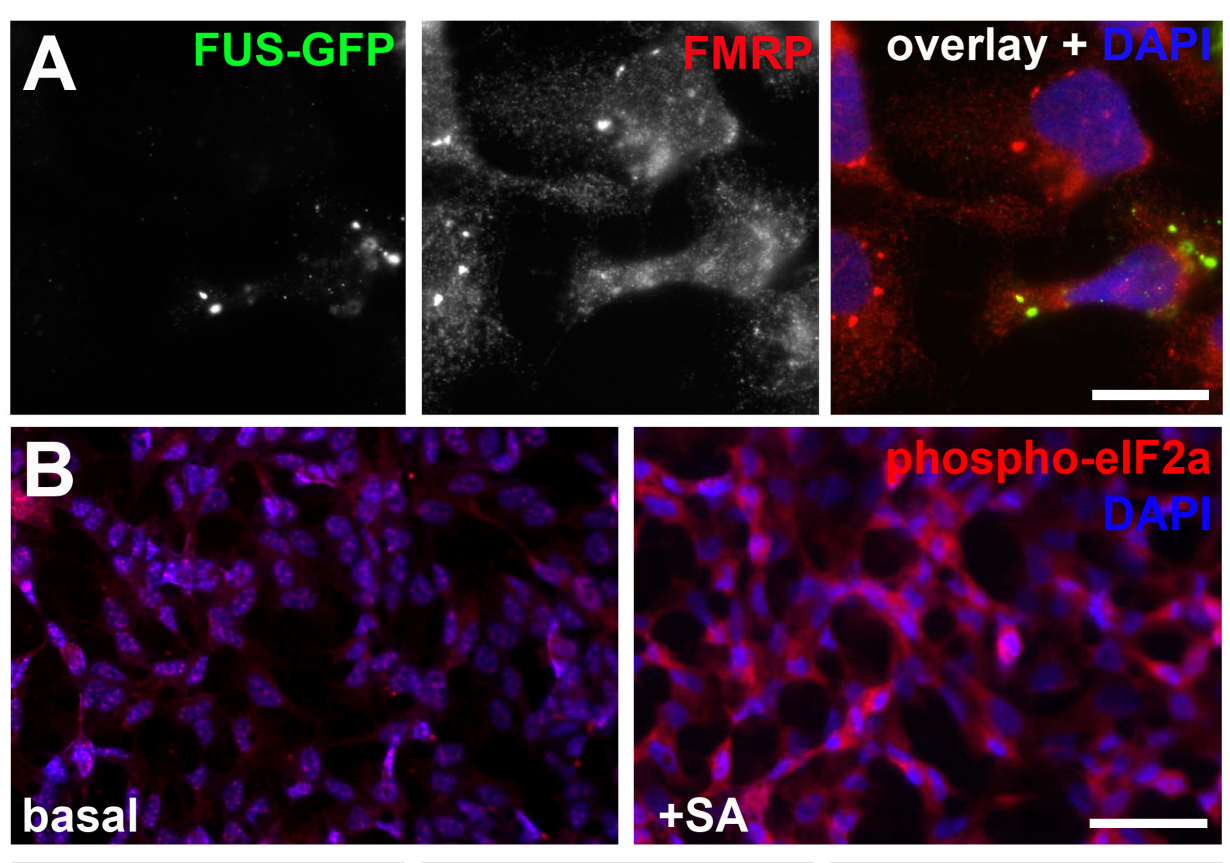
**

**Figure S4.** **Specificity of staining for phospho-eIF2alpha and FMRP antibodies** (A) FMRP, a typical SG protein, is detected in sodium arsenite induced SGs. (B) Level of phospho-eIF2alpha markedly increases upon stress exposure. Scale bars, A - 10 µm, B – 25 µm.


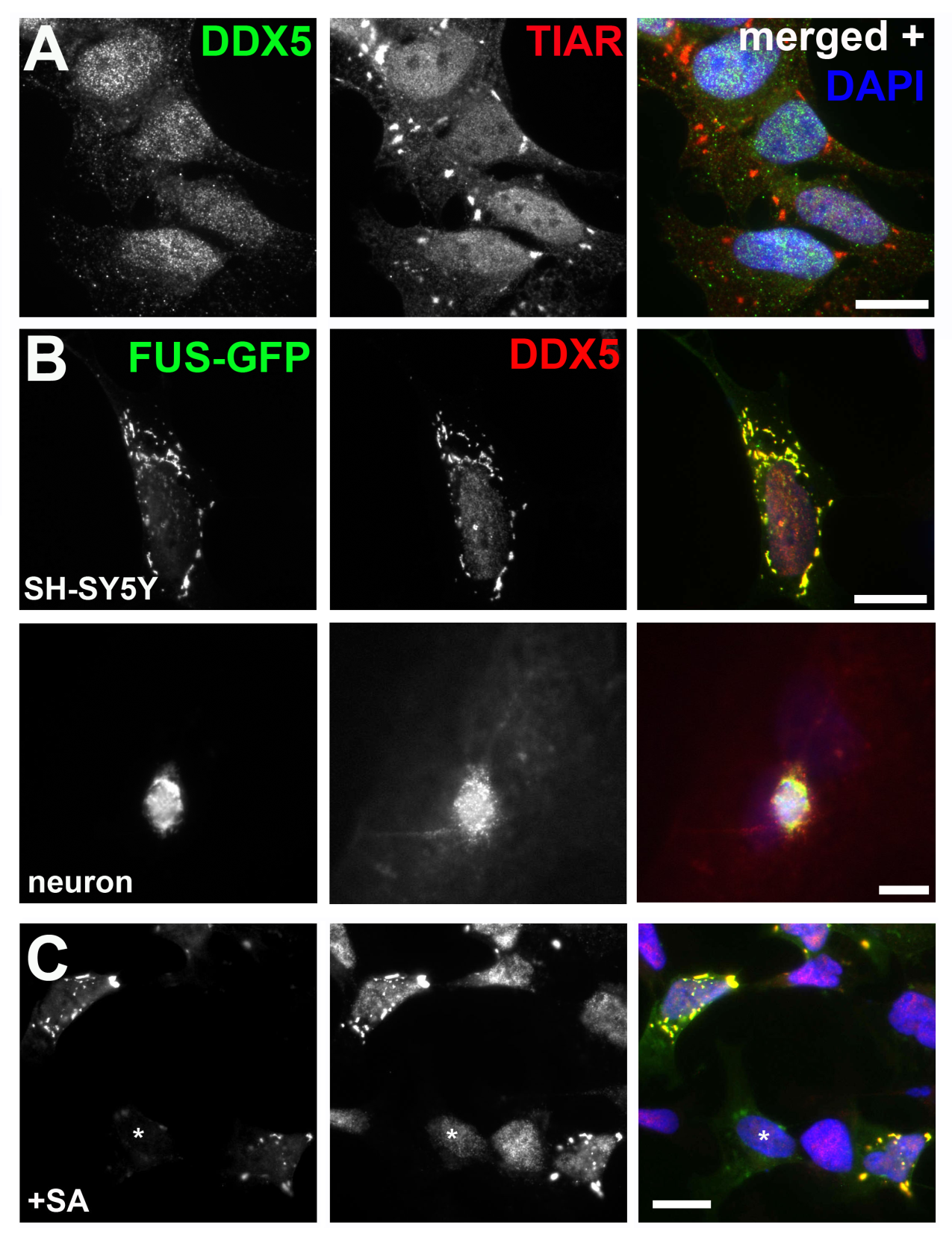


**Figure S5. RNA helicase DDX5 can be used as a selective marker of FAs distinguishing them from SGs.** (A) DDX5 is not recruited to sodium arsenite-induced SGs as revealed by co-staining with anti-DDX5 and anti-TIAR antibodies. (B) FAs formed in SH-SY5Y cells or primary hippocampal neurons expressing GFP-FUS R522G are strongly positive for DDX5. (C) In cells with low level of FUS and lacking FGs/FAs, sodium arsenite treatment induces formation of FUS-positive SGs that are negative for DDX5, as in the cell marked with an asterisk. Scale bars, 10 µm.

**
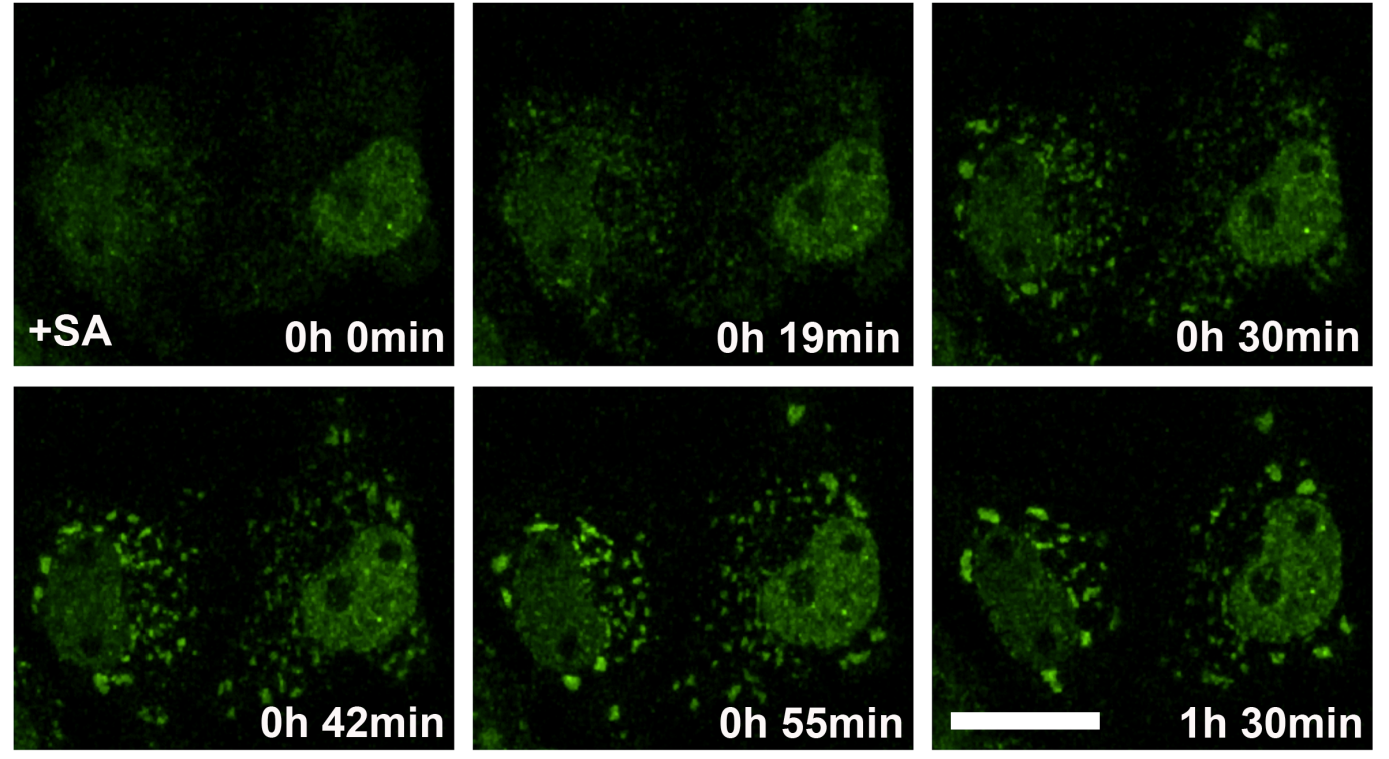
**

**Figure S6.** **Assembly of FUS-positive SGs in SH-SY5Y cells treated with sodium arsenite.** SGs are assembled in the usual way in cells with low FUS levels and its diffuse distribution in the cytoplasm. 24 h after transfection with an expression plasmid encoding GFP-FUS R522G protein sodium arsenite was added to the cell culture (0h 0min time point) and a gallery of images was taken using confocal microscope. See also Supp.VideoS2. Scale bar, 10 µm.

**
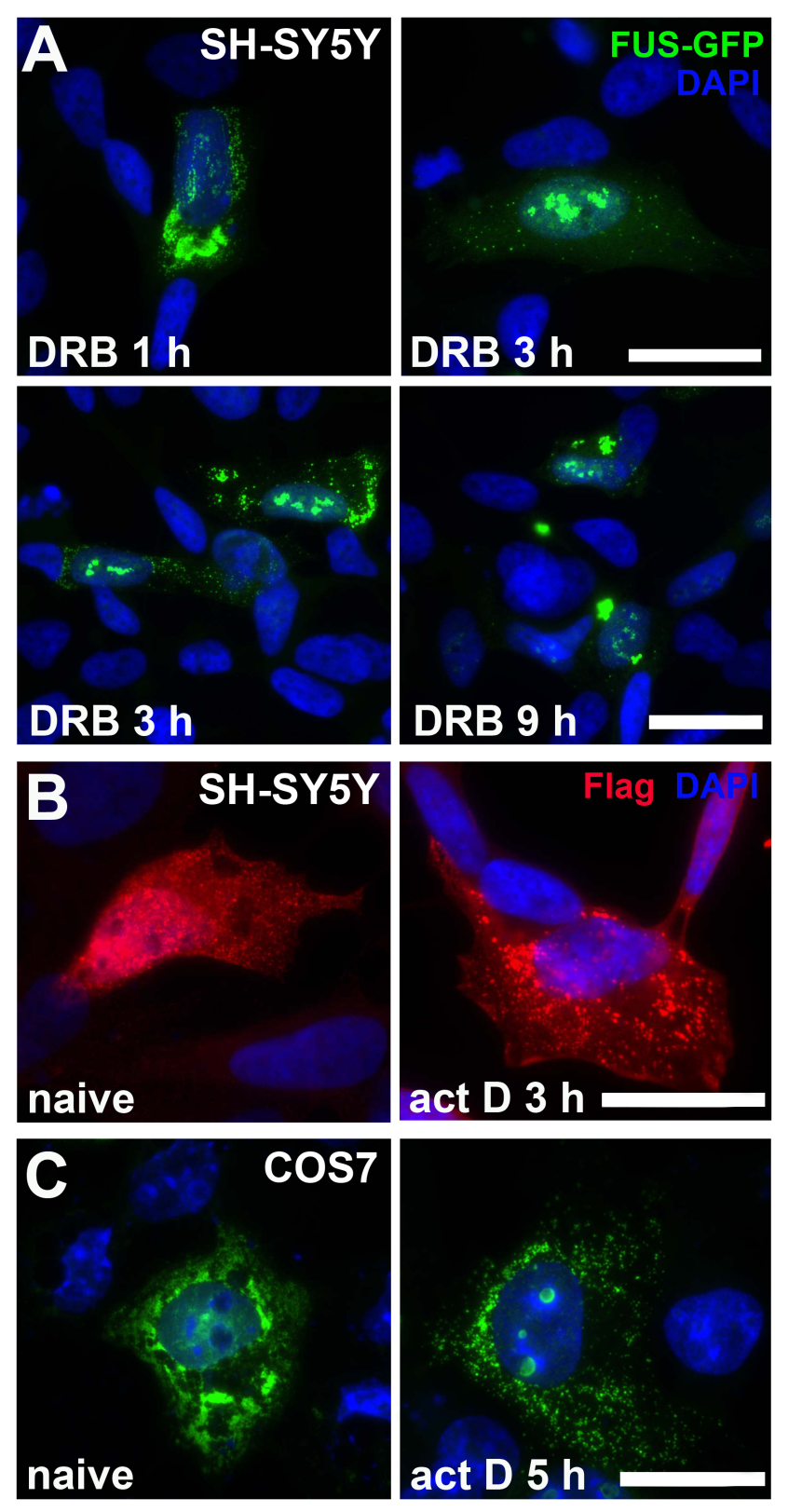
**

**Figure S7. RNA-independent aggregation of GFP or Flag-tagged FUS R522G upon inhibition of transcription in SH-SY5Y cells and COS7 cells.** (A) SH-SY5Y expressing GFP-tagged FUS were treated with a selective DNA polymerase II inhibitor DRB. Note accumulation of FUS in the perinucleolar region typical for DRB, without the formation of nucleolar caps. (B, C) Flag-tagged FUS and GFP-FUS undergo secondary, RNA-independent aggregation in SH-SY5Y and COS7 cells upon actinomycin D exposure. Scale bars, 15 µm.

**
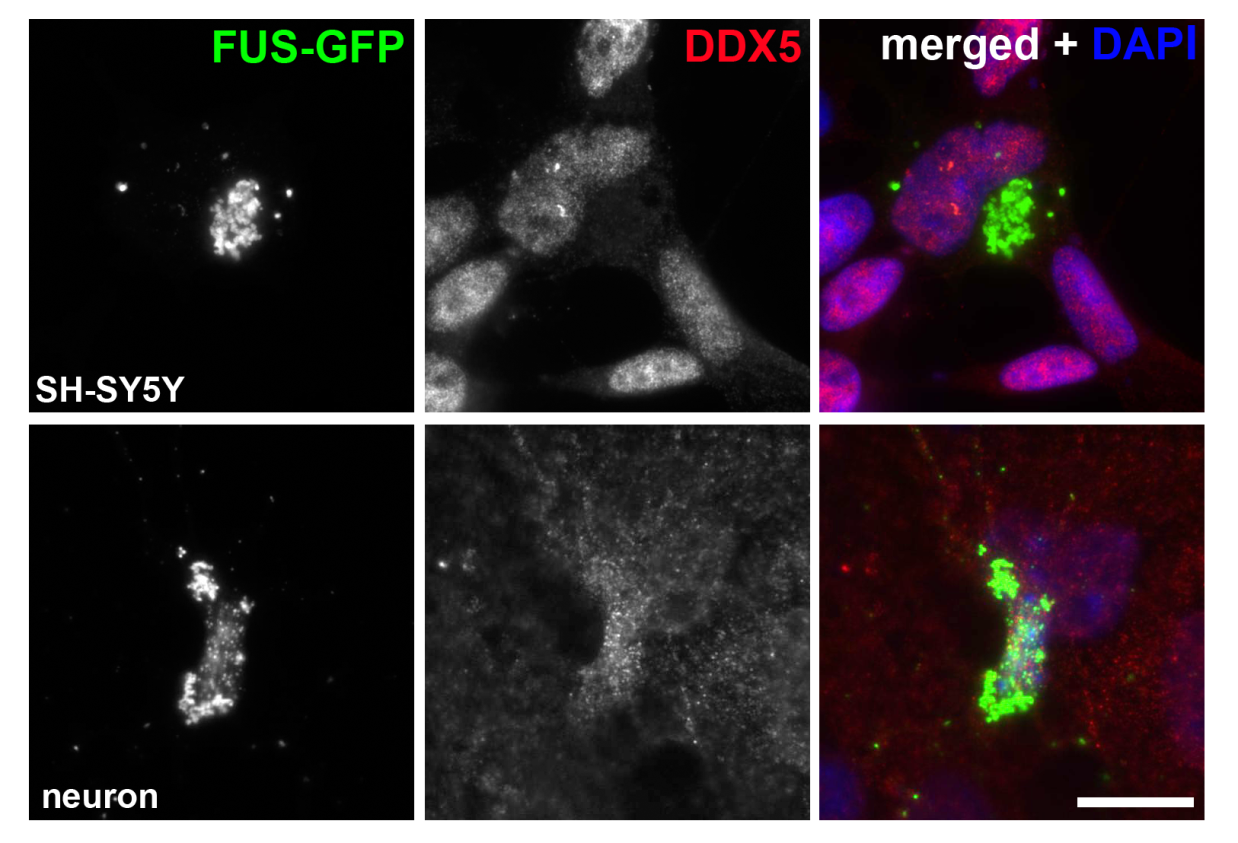
**

**Figure S8. DDX5 does not co-aggregate with RNA-deficient FUS variant lacking C-terminal RNA-binding motifs (NT-RRM).** SH-SY5Y cells or mixed neuronal-glial cultures were fixed 48 h after transfection and immunostained for DDX5. Scale bar, 10 µm.

**Supplementary Video S1.** Assembly of FGs into FAs. COS7 cells were transfected with the expression plasmid encoding GFP-fused FUS R522G, and 12 hours post-transfection time lapse imaging of a cell with the protein at 0:00 time point both diffusely distributed in the cytoplasm and found in FGs was carried out for 6 hours.

**Supplementary Video S2.** Stress-induced assembly of FGs into FAs. COS7 cells were transfected with the expression plasmid encoding GFP-fused FUS R522G, culture medium containing sodium arsenite was added to the cells 24 hours post-transfection, and time lapse imaging of cells containing at 0:00 time point pre-formed FGs in their cytoplasm was carried out for 1.5 hours.

**Supplementary Video S3.** Formation of stress granules in cells with diffuse cytoplasmic distribution of FUS. COS7 cells were transfected with the expression plasmid encoding GFP-fused FUS R522G, culture medium containing sodium arsenite was added to the cells 24 hours post-transfection, and time lapse imaging of cells with the protein at 0:00 time point diffusely distributed in the cytoplasm and displaying no FGs was carried out for 1.5 hours.
